# Germline mutation profiling of breast cancer patients using a non-BRCA sequencing panel

**DOI:** 10.3389/fbinf.2025.1620025

**Published:** 2025-09-02

**Authors:** Sonar Soni Panigoro, Rafika Indah Paramita, Fadilah Fadilah, Septelia Inawati Wanandi, Aisyah Fitriannisa Prawiningrum, Linda Erlina, Wahyu Dian Utari, Ajeng Megawati Fajrin

**Affiliations:** 1 Surgical Oncology Division, Department of Surgery, Faculty of Medicine, Universitas Indonesia, Jakarta, Indonesia; 2 Department of Medical Chemistry, Faculty of Medicine, Universitas Indonesia, Jakarta, Indonesia; 3 Bioinformatics Core Facilities - IMERI, Faculty of Medicine, Universitas Indonesia, Jakarta, Indonesia; 4 Master’s Programme in Biomedical Sciences, Faculty of Medicine, Universitas Indonesia, Jakarta, Indonesia; 5 Department of Biochemistry and Molecular Biology, Faculty of Medicine, Universitas Indonesia, Jakarta, Indonesia; 6 Molecular Biology and Proteomics Core Facilities-IMERI, Faculty of Medicine, Universitas Indonesia, Jakarta, Indonesia

**Keywords:** breast cancer, FASTQ data, next-generation sequencing, non-BRCA sequencing panels, pathogenic mutation

## 1 Introduction

Breast cancer remains the most prevalent form of cancer worldwide. Based on data from the Global Cancer Observatory (GLOBOCAN) in 2020, breast cancer ranks first in the category of new cases of cancer worldwide (11.7%) and ranks fifth as a cause of death (6.9%) (Sung et al., 2021). Mutations in the *BRCA1* and *BRCA2* genes have been extensively studied and are known to be associated with an increased risk of developing the disease (Momozawa et al., 2022). While *BRCA1* and *BRCA2* mutations are well-known germline mutations associated with an increased risk of breast cancer, several other non-*BRCA* genes can also harbor germline mutations linked to breast cancer susceptibility—for example, *TP53*, *PTEN*, *STK11*, *PALB2*, *CHEK2*, *ATM*, *RAD51C*, and *RAD51D* genes (Wang et al., 2021). The aforementioned genes play a crucial role in DNA repair, cell cycle regulation, and the inhibition of tumor formation (Yang et al., 2022). Identifying germline mutations, not only in *BRCA* genes but also in other genes, can have important implications for both affected individuals and their families, allowing for prevention and treatment strategies (Wang et al., 2018; Momozawa et al., 2018). In this cross-sectional study, we aimed to identify non-*BRCA* germline mutations found in breast cancer patients using a less-invasive method that could serve as a biomarker for breast cancer subtyping.

## 2 Methods

### 2.1 Sample collection and DNA purification

A total of 28 female individuals diagnosed with breast cancer participated in this study, and blood samples were obtained from each participant. DNA extraction was conducted using the Genomic DNA Mini Kit® (Geneaid, New Taipei City, Taiwan), following the manufacturer’s instructions. The purity of DNA isolates was assessed by measuring the 260/280 absorbance ratio using a NanoDrop instrument (Thermo Fisher Scientific, Waltham, MA, United States). The quantification of DNA isolates was performed using the Qubit dsDNA HS Assay Kit (Thermo Fisher Scientific, Waltham, MA, United States) on a Qubit® 4.0 Fluorometer (Thermo Fisher Scientific, Waltham, MA, United States).

### 2.2 Library preparation and sequencing

Library preparation was performed utilizing the Illumina AmpliSeq™ Cancer Hotspot Panel v2 (Illumina®, United States). The first step involved the amplification of specific areas within the DNA sample. The amplicons were subsequently subjected to partial digestion using the FuPa reagent. The indexes were ligated using the Ligate program on a thermal cycler. In order to purify the libraries, 30 μL of Agencourt AMPure XP beads (Beckman Coulter™, United States) was added to the reaction mixtures.

Amplification techniques were implemented in order to ensure a sufficient quantity of the libraries. The second round of purification was subsequently performed twice to remove high molecular weight DNA and excess primers, using Agencourt AMPure XP beads (Beckman Coulter™, United States). The libraries were diluted to a final loading concentration and subsequently subjected to sequencing utilizing the Illumina MiSeq technology. Sequencing yielded paired-end libraries in FASTQ format, with a read length of 150 base pairs (bp) for both ends. The data sequences have been deposited in the Sequence Read Archive (SRA) database under BioProject accession number PRJNA998562.

### 2.3 Quality control and data trimming

Quality control of the FASTQ data was conducted to evaluate the quality of each sample’s raw reads. FastQC software (Andrews, 2010) was used to perform FASTQ quality assessment. The total number of raw bases and Q30 percentage were determined using the q30 Python programs (https://github.com/dayedepps/q30/tree/master). If the quality of sequence reads was poor, quality-improvement steps were taken. Trimmomatic (Bolger et al., 2014) was used to trim the low-quality reads and remove adapters (ILLUMINACLIP: NexteraPE-PE.FA: 2:30:10, LEADING: 3, TRAILING: 3, SLIDINGWINDOW: 4:15, and MINLEN: 35). Read alignment was performed using BWA-MEM (Li and Durbin, 2009), with GRCh38. p13 as the human reference genome. After alignment, the amplicon mean depth, coverage uniformity, and the percentage of on-target rate were calculated using an in-house script containing Mosdepth (Pedersen and Quinlan, 2018), SAMtools (Danecek et al., 2021), and BEDTools (Danecek et al., 2021) software. The command-line scripts used to calculate the Q30 percentage, amplicon mean depth, coverage uniformity, and the percentage of on-target rate are provided in Supplementary Material.

### 2.4 Variant calling analysis

Variant calling analysis was performed to find likely pathogenic and pathogenic variants in all samples. The workflow followed the methods described by Panigoro et al. (2022) and included read alignment using BWA (Li and Durbin, 2009), SAM-to-BAM conversion using SAMTools (Li et al., 2009), variant calling using GATK (McKenna et al., 2010), and variant annotation using SnpEff and SnpSift (Cingolani et al., 2012). Germline variant classification was conducted using VarSome (Kopanos et al., 2019) (https://varsome.com/), which applies the ACMG classification guidelines. A total score is computed by summing the points from pathogenic rules and subtracting the points from benign rules. The total score is then compared with predefined thresholds to determine the final verdict: pathogenic if greater than or equal to 10, likely pathogenic if between 6 and 9 inclusive, uncertain significance if between 0 and 5, likely benign if between −6 and −1, and benign if less than or equal to −7. The command-line scripts used for variant calling analysis are available on GitHub: https://github.com/fikaparamita04/variant-calling. The most frequently observed pathogenic variants were visualized using MutationMapper (Vohra and Biggin, 2013) (https://www.cbioportal.org/mutation_mapper).

## 3 Data analysis

### 3.1 Patients

We successfully collected blood samples from 28 patients diagnosed with breast cancer at Cipto Mangunkusumo National Hospital, Jakarta. The patients ranged in age from 40 to 71 years (Table 1). The patients were categorized into four subtypes, namely, Luminal A, Luminal B, HER2-positive and triple-negative breast cancer (TNBC), with the total number of patients being 8, 9, 7, and 4, respectively. Among the subjects, four patients were diagnosed at stage IV.

**TABLE 1:** Descriptive information of the patients.

Sample ID	Age	Stage	Molecular subtype
BC_2_CMNH_19	41	IIIC	Luminal B
BC_6_CMNH_19	43	IIA	Luminal A
BC_11_CMNH_19	47	IIIA	TNBC
BC_12_CMNH_19	48	IIA	Luminal A
BC_13_CMNH_19	48	IIA	Luminal A
BC_14_CMNH_19	61	IV	TNBC
BC_15_CMNH_19	40	IIIB	HER2-positive
BC_16_CMNH_19	59	IIIB	Luminal B
BC_17_CMNH_19	40	IIIA	Luminal A
BC_18_CMNH_19	43	IIIC	HER2-positive
BC_20_CMNH_19	41	IIIB	HER2-positive
BC_21_CMNH_19	44	IIA	Luminal A
BC_22_CMNH_19	52	IIB	Luminal A
BC_23_CMNH_19	71	IIB	Luminal B
BC_25_CMNH_19	52	IV	Luminal A
BC_28_CMNH_19	54	IIA	Luminal B
BC_29_CMNH_19	65	IIIC	Luminal B
BC_30_CMNH_19	54	IIB	HER2-positive
BC_31_CMNH_19	60	IIB	Luminal A
BC_32_CMNH_19	48	IIIA	TNBC
BC_33_CMNH_19	41	IIIC	TNBC
BC_34_CMNH_19	41	IIB	Luminal B
BC_35_CMNH_19	60	IV	HER2-positive
BC_36_CMNH_19	66	IIIB	Luminal B
BC_38_CMNH_19	62	IIIB	HER2-positive
BC_39_CMNH_19	50	IIB	Luminal B
BC_40_CMNH_19	59	IV	Luminal B
BC_41_CMNH_19	42	IIIB	HER2-positive

### 3.2 Quality control of FASTQ data

Raw FASTQ data were quality-checked to ensure high sequencing quality. Given that this was a targeted sequencing study, we evaluated the Q30 percentage, average amplicon depth, coverage uniformity, and target level percentage (Table 2). The Q30 result of 97.71% ± 0.44 indicates high-quality sequencing. The average amplicon depth was also strong, with a score of 1,076 ± 256.36, although variability between samples was relatively high. Coverage uniformity, which measures how evenly sequencing reads are distributed across the genome or a specific region of interest, was excellent across all samples. All samples showed a coverage uniformity score of 1, or close to 1 (0.9901 ± 0.01), indicating that all target bases were covered to the same extent, without regions of significantly higher or lower read depth. The target-level percentage, which reflects the proportion of bases within the targeted regions that were successfully sequenced, was also high at 95.57% ± 0.57. The raw data files in FASTQ format have been archived in the BioProject database under accession number PRJNA998562. These data may serve as a potentially valuable resource for screening gene mutation markers in breast cancer and could aid in predicting treatment efficacy related to specific mutations.

**TABLE 2:** Descriptive information of targeted sequencing evaluations.

Sample ID	Q30 (%)	Amplicon mean depth	Coverage uniformity	Percentage of the on-target rate
BC_2_CMNH_19	97.22	612	0.9887	95.87
BC_6_CMNH_19	97.34	1,118	1	95.02
BC_11_CMNH_19	98.17	746	0.9943	95.80
BC_12_CMNH_19	97.25	1,443	0.9943	95.53
BC_13_CMNH_19	97.04	1,233	0.9887	95.68
BC_14_CMNH_19	98.11	975	0.9828	96.50
BC_15_CMNH_19	97.24	736	0.9943	96.16
BC_16_CMNH_19	97.22	1,186	0.9943	94.56
BC_17_CMNH_19	97.08	1,193	0.9943	95.15
BC_18_CMNH_19	97.22	1,411	0.9943	95.40
BC_20_CMNH_19	97.24	1,245	0.9943	95.25
BC_21_CMNH_19	98.20	998	0.9828	95.51
BC_22_CMNH_19	98.15	607	0.9943	96.12
BC_23_CMNH_19	98.03	1,289	0.9943	95.30
BC_25_CMNH_19	98.04	951	0.9943	95.12
BC_28_CMNH_19	97.21	1,525	0.9887	95.75
BC_29_CMNH_19	98.15	919	0.9828	95.89
BC_30_CMNH_19	98.19	1,234	0.9782	95.57
BC_31_CMNH_19	98.07	942	1	95.52
BC_32_CMNH_19	97.92	1,341	0.9943	95.86
BC_33_CMNH_19	98.19	1,067	0.9943	95.51
BC_34_CMNH_19	97.36	1,232	0.9887	95.64
BC_35_CMNH_19	97.26	1,344	0.9443	93.63
BC_36_CMNH_19	98.18	791	0.9782	95.78
BC_38_CMNH_19	97.87	766	1	96.51
BC_39_CMNH_19	97.92	895	1	95.52
BC_40_CMNH_19	97.92	1,321	0.9943	95.61
BC_41_CMNH_19	98.12	1,013	0.9943	96.06

### 3.3 Variant calling analysis

**FIGURE 1:**
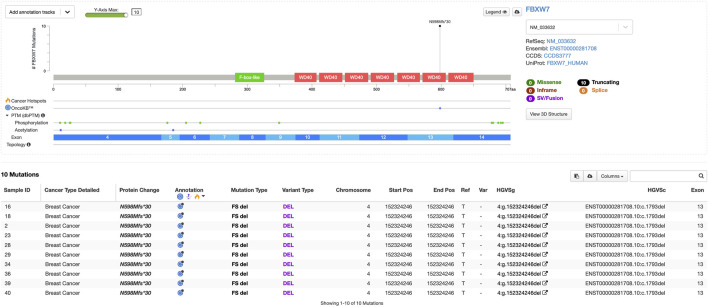
Lollipop plot of germline pathogenic variants in the *FBXW7* gene (visualization using MutationMapper).

We found the highest germline frameshift mutation in the *FBXW7* gene (4:g.152324246del), with a frequency of approximately 35.7%. This variant was predicted as likely pathogenic by VarSome (prediction score = 9), due to the loss of protein functions (Figure 1). Interestingly, this mutation was found in all Luminal B and one HER2-positive patient. FBXW7 is a tumor suppressor that modulates the degradation of oncogenic substrates, including c-Jun, c-Myc, the Notch1 intracellular domain (ICD), and cyclin E, by acting as the substrate recognition protein within the Skp1–Cullin–F-box (SCF) ubiquitin ligase complex. Deletion of chromosome 4q3, which encompasses FBXW7, occurs in approximately 30% of primary breast tumors (Meyer et al., 2020).

In line with previous studies, the deletion mutation in the FBXW7 gene closely resembles the human breast cancer luminal B subtype, characterized by ERα+, PR-, and elevated Ki67 staining (Meyer et al., 2020). Furthermore, Luminal B tumors exhibit the lowest FBXW7 mRNA expression among breast cancer subtypes. Lower FBXW7 expression is associated with a high Ki-67 labeling index and positive cyclin E protein expression, both indicators of proliferation. Breast cancer patients with the greatest FBXW7 gene expression have a longer disease-free survival rate (Yeh et al., 2018). The process by which FBXW7 regulates breast cancer growth, cell cycle, and metastasis involves many signaling pathways and gene interactions. For example, FBXW7-deficient breast tumors inhibit the NF-κB signaling pathway, which normally involves E3 ubiquitin ligase binding and degradation. This inhibition results in enhanced NF-κB DNA-binding activity, promoting tumor development and metastasis (Chen et al., 2023).

As shown in Figure 1, the mutation found in the *FBXW7* gene is also reported in the OncoKB database (Chakravarty et al., 2017). According to the database, FBXW7 N598Mfs*30 is a truncating mutation in a tumor suppressor gene and is, therefore, considered likely oncogenic. There is promising scientific and anecdotal clinical evidence supporting the use of lunresertib and camonsertib in patients with FBXW7-mutated solid tumors. Lunresertib is an orally available, small-molecule PKMYT1 inhibitor, while camonsertib is an orally available, small-molecule ATR inhibitor. In the Phase I MYTHIC trial of lunresertib plus camonsertib in patients with advanced tumors harboring CCNE1 amplifications, FBXW7 deleterious mutations, or PPP2R1A deleterious mutations, the lunresertib + camonsertib cohort (n = 59 [n = 17 endometrial; n = 13 colorectal; n = 11 ovarian; n = 3, breast; n = 3, lung; n = 12, other]) showed an overall response rate of 23.6% among all evaluable patients across tumor types (n = 55) (Yap et al., 2023). However, future studies with larger patient populations and integration of multi-omics approaches are needed for precise subtyping and personalized therapy. We hope that our small contribution can help advance precision therapy for breast cancer, particularly in Indonesia.

## Data Availability

The datasets presented in this study can be found in online repositories. The names of the repository/repositories and accession number(s) can be found at https://www.ncbi.nlm.nih.gov/, PRJNA998562.
