# Supplementary material for: Germline mutation profiling of breast cancer patients using a non-BRCA sequencing panel
Source: Front Bioinform. 2025 Sep 2;5:1620025. doi: 10.3389/fbinf.2025.1620025 (PMC12436446; doi:10.3389/fbinf.2025.1620025)
Supplement: Supplementary file 1 [file Supplementaryfile1.docx]

Supplementary Material

# Supplementary File 1

The command-line script used:

**Q30 percentage** using q30 Python programs (<https://github.com/dayedepps/q30/tree/master>)

python q30.py <fastq_file_name>

**Amplicon mean depth** using Mosdepth and Bedtools

mosdepth --by <path/hglft_genome_42ffe_8d7520.bed> <output_prefix_file_name> -t 10 <bam_file>

bedtools coverage -a <regions.bed.gz_file> -b <bam_file> -d > <output_perBaseDepth.bedgraph_file>

**Coverage uniformity**

awk -v counter=0 '{if($5>$meanDepth*0.2) counter++} END{print counter/NR}' <perBaseDepth.bedgraph_file>

**Percentage of on target rate** using Samtools and Bedtools

totalReads=$(samtools view <bam_file> | wc -l)

onTargetReads=$(bedtools intersect -a <bam_file> -b <regions.bed.gz_file> -u | samtools view | wc -l)

echo "" | awk -v OFS="" -v total=$totalReads -v target=$onTargetReads '{print (target/total)*100,"%"}'

hglft_genome_42ffe_8d7520.bed

1 43349297 43349415 CHP2_MPL_1 1

1 114709564 114709648 CHP2_NRAS_3 1

1 114713883 114713963 CHP2_NRAS_2 1

1 114716068 114716153 CHP2_NRAS_1 1

2 29209706 29209814 CHP2_ALK_2 1

2 29220741 29220863 CHP2_ALK_1 1

2 208248379 208248482 CHP2_IDH1_1 1

2 211424179 211424265 CHP2_ERBB4_8 1

2 211665326 211665455 CHP2_ERBB4_7 1

2 211712074 211712185 CHP2_ERBB4_6 1

2 211713563 211713690 CHP2_ERBB4_5 1

2 211722408 211722514 CHP2_ERBB4_4 1

2 211725039 211725142 CHP2_ERBB4_3 1

2 211787994 211788081 CHP2_ERBB4_2 1

2 211947350 211947444 CHP2_ERBB4_1 1

3 10142081 10142170 CHP2_VHL_1 1

3 10146502 10146622 CHP2_VHL_2 1

3 10149734 10149843 CHP2_VHL_3 1

3 37025716 37025842 CHP2_MLH1_1 1

3 41224538 41224656 CHP2_CTNNB1_1 1

3 179198987 179199093 CHP2_PIK3CA_1 1

3 179199143 179199247 CHP2_PIK3CA_2 1

3 179203676 179203782 CHP2_PIK3CA_3 1

3 179209617 179209737 CHP2_PIK3CA_4 1

3 179210113 179210198 CHP2_PIK3CA_5 1

3 179210281 179210372 CHP2_PIK3CA_6 1

3 179218235 179218317 CHP2_PIK3CA_7 1

3 179220999 179221130 CHP2_PIK3CA_8 1

3 179230030 179230108 CHP2_PIK3CA_9 1

3 179234208 179234309 CHP2_PIK3CA_10 1

3 179234352 179234449 CHP2_PIK3CA_11 1

4 1801824 1801926 CHP2_FGFR3_1 1

4 1804354 1804460 CHP2_FGFR3_2 1

4 1806106 1806203 CHP2_FGFR3_3 1

4 1806584 1806672 CHP2_FGFR3_4 1

4 1807154 1807279 CHP2_FGFR3_5 1

4 54274814 54274936 CHP2_PDGFRA_1 1

4 54277934 54278028 CHP2_PDGFRA_2 1

4 54278371 54278486 CHP2_PDGFRA_3 1

4 54285857 54285987 CHP2_PDGFRA_4 1

4 54695488 54695618 CHP2_KIT_1 1

4 54725991 54726080 CHP2_KIT_2 1

4 54727251 54727347 CHP2_KIT_3 1

4 54727409 54727529 CHP2_KIT_4 1

4 54728004 54728113 CHP2_KIT_5 1

4 54729330 54729396 CHP2_KIT_6 1

4 54731270 54731358 CHP2_KIT_7 1

4 54733114 54733192 CHP2_KIT_8 1

4 54736507 54736585 CHP2_KIT_9 1

4 55079921 55080041 CHP2_KDR_9 1

4 55080083 55080204 CHP2_KDR_8 1

4 55087608 55087693 CHP2_KDR_7 1

4 55088911 55089001 CHP2_KDR_6 1

4 55094809 55094892 CHP2_KDR_5 1

4 55096277 55096381 CHP2_KDR_4 1

4 55106785 55106904 CHP2_KDR_3 1

4 55113407 55113488 CHP2_KDR_2 1

4 55114071 55114192 CHP2_KDR_1 1

4 152324258 152324340 CHP2_FBXW7_5 1

4 152326125 152326217 CHP2_FBXW7_4 1

4 152328203 152328325 CHP2_FBXW7_3 1

4 152329700 152329774 CHP2_FBXW7_2 1

4 152337749 152337871 CHP2_FBXW7_1 1

5 112838174 112838265 CHP2_APC_1 1

5 112838860 112838969 CHP2_APC_2 1

5 112839446 112839571 CHP2_APC_3 1

5 112839618 112839746 CHP2_APC_4 1

5 112839870 112840006 CHP2_APC_5 1

5 112840043 112840165 CHP2_APC_6 1

5 112840223 112840338 CHP2_APC_7 1

5 150054030 150054129 CHP2_CSF1R_2 1

5 150073428 150073510 CHP2_CSF1R_1 1

5 171410497 171410612 CHP2_NPM1_1 1

7 55143351 55143433 CHP2_EGFR_1 1

7 55154099 55154226 CHP2_EGFR_2 1

7 55165269 55165360 CHP2_EGFR_3 1

7 55173942 55174036 CHP2_EGFR_4 1

7 55174718 55174847 CHP2_EGFR_5 1

7 55181272 55181397 CHP2_EGFR_6 1

7 55181429 55181552 CHP2_EGFR_7 1

7 55191814 55191935 CHP2_EGFR_8 1

7 116699561 116699647 CHP2_MET_1 1

7 116700101 116700216 CHP2_MET_2 1

7 116763077 116763197 CHP2_MET_3 1

7 116771824 116771943 CHP2_MET_4 1

7 116777373 116777488 CHP2_MET_5 1

7 116783353 116783438 CHP2_MET_6 1

7 129205222 129205347 CHP2_SMO_1 1

7 129206116 129206222 CHP2_SMO_2 1

7 129206496 129206578 CHP2_SMO_3 1

7 129210428 129210522 CHP2_SMO_4 1

7 129211658 129211771 CHP2_SMO_5 1

7 140753302 140753421 CHP2_BRAF_2 1

7 140781591 140781715 CHP2_BRAF_1 1

7 148811614 148811699 CHP2_EZH2_1 1

8 38424622 38424736 CHP2_FGFR1_2 1

8 38428333 38428457 CHP2_FGFR1_1 1

9 5073729 5073857 CHP2_JAK2_1 1

9 21970941 21971067 CHP2_CDKN2A_2 1

9 21971091 21971220 CHP2_CDKN2A_1 1

9 77794459 77794582 CHP2_GNAQ_1 1

9 130862907 130862991 CHP2_ABL1_1 1

9 130872055 130872143 CHP2_ABL1_2 1

9 130872892 130873030 CHP2_ABL1_3 1

9 130874920 130875018 CHP2_ABL1_4 1

9 136496312 136496433 CHP2_NOTCH1_3 1

9 136503310 136503427 CHP2_NOTCH1_2 1

9 136504885 136504995 CHP2_NOTCH1_1 1

10 43113618 43113734 CHP2_RET_1 1

10 43114426 43114562 CHP2_RET_2 1

10 43118346 43118445 CHP2_RET_3 1

10 43120098 43120239 CHP2_RET_4 1

10 43121866 43121985 CHP2_RET_5 1

10 87864450 87864543 CHP2_PTEN_1 1

10 87925501 87925617 CHP2_PTEN_2 1

10 87933056 87933163 CHP2_PTEN_3 1

10 87952047 87952175 CHP2_PTEN_4 1

10 87957746 87957863 CHP2_PTEN_5 1

10 87957909 87958023 CHP2_PTEN_6 1

10 87960938 87960990 CHP2_PTEN_7 1

10 87961027 87961143 CHP2_PTEN_8 1

10 121498438 121498531 CHP2_FGFR2_4 1

10 121515207 121515321 CHP2_FGFR2_3 1

10 121519903 121520030 CHP2_FGFR2_2 1

10 121520093 121520199 CHP2_FGFR2_1 1

11 533812 533930 CHP2_HRAS_2 1

11 534220 534308 CHP2_HRAS_1 1

11 108247038 108247138 CHP2_ATM_1 1

11 108249088 108249164 CHP2_ATM_2 1

11 108252788 108252891 CHP2_ATM_3 1

11 108267204 108267298 CHP2_ATM_4 1

11 108284356 108284453 CHP2_ATM_5 1

11 108299729 108299829 CHP2_ATM_6 1

11 108301635 108301740 CHP2_ATM_7 1

11 108302903 108302976 CHP2_ATM_8 1

11 108310175 108310233 CHP2_ATM_9 1

11 108330188 108330266 CHP2_ATM_10 1

11 108333907 108333957 CHP2_ATM_11 1

11 108335004 108335089 CHP2_ATM_12 1

11 108335796 108335901 CHP2_ATM_13 1

11 108347288 108347417 CHP2_ATM_14 1

11 108354822 108354905 CHP2_ATM_15 1

11 108365315 108365413 CHP2_ATM_16 1

11 108365459 108365558 CHP2_ATM_17 1

12 25225615 25225724 CHP2_KRAS_3 1

12 25227326 25227430 CHP2_KRAS_2 1

12 25245252 25245370 CHP2_KRAS_1 1

12 112450314 112450424 CHP2_PTPN11_1 1

12 112489031 112489157 CHP2_PTPN11_2 1

12 120993568 120993656 CHP2_HNF1A_1 1

12 120994207 120994296 CHP2_HNF1A_2 1

13 28018442 28018526 CHP2_FLT3_4 1

13 28028138 28028242 CHP2_FLT3_3 1

13 28034090 28034211 CHP2_FLT3_2 1

13 28035956 28036047 CHP2_FLT3_1 1

13 48345087 48345176 CHP2_RB1_1 1

13 48349003 48349119 CHP2_RB1_2 1

13 48367465 48367588 CHP2_RB1_3 1

13 48368461 48368575 CHP2_RB1_4 1

13 48379617 48379738 CHP2_RB1_5 1

13 48381389 48381469 CHP2_RB1_6 1

13 48452969 48453042 CHP2_RB1_7 1

13 48459691 48459798 CHP2_RB1_8 1

13 48463710 48463796 CHP2_RB1_9 1

13 48465013 48465096 CHP2_RB1_10 1

14 104775096 104775182 CHP2_AKT1_2 1

14 104780108 104780246 CHP2_AKT1_1 1

15 90088592 90088722 CHP2_IDH2_1 1

16 68801699 68801794 CHP2_CDH1_1 1

16 68812121 68812248 CHP2_CDH1_2 1

16 68813296 68813399 CHP2_CDH1_3 1

17 7670605 7670717 CHP2_TP53_8 1

17 7673697 7673833 CHP2_TP53_7 1

17 7674190 7674294 CHP2_TP53_6 1

17 7674862 7674980 CHP2_TP53_5 1

17 7675034 7675165 CHP2_TP53_4 1

17 7675198 7675283 CHP2_TP53_3 1

17 7676032 7676167 CHP2_TP53_2 1

17 7676535 7676642 CHP2_TP53_1 1

17 39723959 39724087 CHP2_ERBB2_1 1

17 39724700 39724808 CHP2_ERBB2_2 1

17 39725071 39725200 CHP2_ERBB2_3 1

18 51048729 51048843 CHP2_SMAD4_1 1

18 51049186 51049307 CHP2_SMAD4_2 1

18 51054820 51054932 CHP2_SMAD4_3 1

18 51058181 51058308 CHP2_SMAD4_4 1

18 51059881 51059991 CHP2_SMAD4_5 1

18 51065444 51065561 CHP2_SMAD4_6 1

18 51067029 51067149 CHP2_SMAD4_7 1

18 51076658 51076749 CHP2_SMAD4_8 1

18 51078288 51078404 CHP2_SMAD4_9 1

19 1206978 1207105 CHP2_STK11_1 1

19 1220311 1220451 CHP2_STK11_2 1

19 1220481 1220604 CHP2_STK11_3 1

19 1221237 1221333 CHP2_STK11_4 1

19 1223015 1223145 CHP2_STK11_5 1

19 3118883 3118975 CHP2_GNA11_1 1

19 17834807 17834925 CHP2_JAK3_3 1

19 17837177 17837265 CHP2_JAK3_2 1

19 17843325 17843416 CHP2_JAK3_1 1

20 37403263 37403366 CHP2_SRC_1 1

20 58909341 58909449 CHP2_GNAS_1 1

20 58909507 58909617 CHP2_GNAS_2 1

22 23791766 23791877 CHP2_SMARCB1_1 1

22 23801013 23801124 CHP2_SMARCB1_2 1

22 23803290 23803411 CHP2_SMARCB1_3 1

22 23834072 23834204 CHP2_SMARCB1_4 1
